# Light Management with Natural Materials: From Whiteness to Transparency

**DOI:** 10.1002/adma.202001215

**Published:** 2020-07-23

**Authors:** Gianni Jacucci, Lukas Schertel, Yating Zhang, Han Yang, Silvia Vignolini

**Affiliations:** ^1^ Department of Chemistry University of Cambridge Lensfield Road Cambridge CB2 1EW UK

**Keywords:** bioinspiration, disordered photonics, nanocellulose, transparent wood, whiteness

## Abstract

The possibility of structuring material at the nanoscale is essential to control light–matter interactions and therefore fabricate next‐generation paints and coatings. In this context, nature can serve not only as a source of inspiration for the design of such novel optical structures, but also as a primary source of materials. Here, some of the strategies used in nature to optimize light–matter interaction are reviewed and some of the recent progress in the production of optical materials made solely of plant‐derived building blocks is highlighted. In nature, nano‐ to micrometer‐sized structured materials made from biopolymers are at the origin of most of the light‐transport effects. How natural photonic systems manage light scattering and what can be learned from plants and animals to produce photonic materials from biopolymers are discussed. Tuning the light‐scattering properties via structural variations allows a wide range of appearances to be obtained, from whiteness to transparency, using the same renewable and biodegradable building blocks. Here, various transparent and white cellulose‐based materials produced so far are highlighted.

## Graphical abstract

A wide range of strategies is used to manage light propagation in plants and animals to produce transparent and white appearances. Inspired by nature, various transparent (low‐scattering) and opaque (high‐scattering) cellulose‐based materials have been developed. The possibility to structure materials at the nanoscale is essential to control light–matter interaction and enables the fabrication of next‐generation optical materials made of renewable biopolymers.

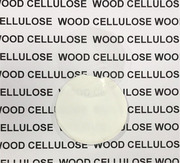

## Introduction

Scattering is the optical effect at the basis of light propagation in non‐absorbing media. This phenomenon occurs when light encounters refractive index inhomogeneities that can be defined as scattering centers or, in the case of granular media, scattering particles.^[^ ^1^, ^2^, ^3^, ^4^, ^5^ ^]^ At every scattering event, light deviates from its initial trajectory depending on the geometrical characteristics and the refractive index of the scatterer (parameters that are described by the scattering cross‐section).^[^ ^1^, ^5^ ^]^

Non‐absorbing homogeneous materials scatter (reflect) light only at their interfaces (surfaces). Therefore, their appearance is determined by the refractive index contrast between the external medium and the homogeneous system. Conversely, for inhomogeneous materials, light transport is a bulk phenomenon. Therefore, their appearance results from the interplay between: 1) the single‐scatterer properties as the scattering cross‐section; 2) the ensemble properties as the filling fraction (i.e., the volume percentage occupied by the scatterers), the average orientation of the scatterers, and the structure factor (i.e., the spatial organization of the scatterers).^[^ ^6^, ^7^ ^]^

**Figure 1:**
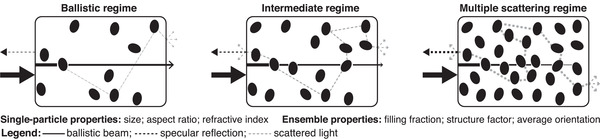
Illustration of different light‐propagation regimes in disordered media: from ballistic to multiple scattering. For simplicity, the transition between different regimes is depicted by varying the filling fraction of the system. Ballistic beam, specular reflection, and scattered light are represented by solid black, dotted black, and dotted gray lines, respectively. Line thickness qualitatively represents the difference in intensity between ballistic and scattered light in every regime.

The appearance of a disordered material (which is defined as system where the structure factor does not exhibit long‐range order) is directly determined by the optical thickness (OT). This parameter is defined as the ratio between the physical thickness of a medium and the transport mean free path, that is, the average distance over which light loses memory of its initial direction. In fact, light transport can undergo different regimes when propagating in disordered media: from ballistic propagation, where most of the intensity is transmitted in the same direction of the incoming beam, to multiple scattering, where the initial propagation direction is completely scrambled (**Figure** **1**). In the ballistic regime, at low OT, light propagation is almost unperturbed, resulting in a transparent appearance as for homogeneous media; in contrast in the multiple scattering, for high OT (>8),^[^ ^8^, ^9^ ^]^ the material is opaque white. Systems in the intermediate scattering regime exhibit interesting optical properties as they strongly affect the light propagation direction while showing high transmission values. This property is called haze.

In this progress report article, we first describe the strategies used by nature to manage light propagation in biological systems to either produce white or transparent appearances. Then, we review the recently developed strategies for fabricating cellulose‐based optical materials to meet the demand for more sustainable, biocompatible products. Finally, an overview of the applications for bio‐inspired materials in both transparent, low‐scattering and opaque, high‐scattering systems is presented.

## Learning from Nature

In this section, we provide an overview of some of the most striking strategies found in nature to manage different regimes of light propagation.

### Minimized Scattering: Transparency in Nature

To minimize scattering, and therefore achieve high transparency, it is necessary to minimize not only refractive index inhomogeneities in the bulk of material but also light scattering at its interfaces (i.e., by anti‐reflection coatings).

**Figure 2:**
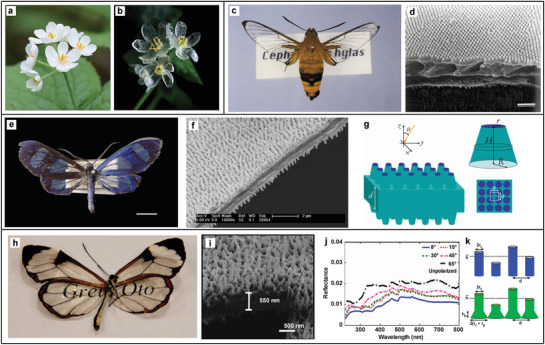
Examples of transparent materials in nature. Photograph of the petals of *Diphylleia grayi* on a dry day (a) and on a rainy day (b). a,b) Reproduced with permission.^[^ ^10^ ^]^ Copyright 2019, the Royal Society of Chemistry. c) Photograph of the hawkmoth *Cephonodes hylas*. d) Scanning electron microscopy (SEM) image of the transparent part of the *Cephonodes* wing. The top side presenting the regular‐hexagonal packing of pillars. Scale bar: 1 μm. c,d) Reproduced with permission.^[^ ^12^ ^]^ Copyright 2019, BioOne. e) Photograph of the moth *Cacostatia ossa*. Scale bar 0.5 cm. f) SEM image of the wing of *Cacostatia ossa*: view of the wing surface at oblique incidence. g) A 3D model of the wing with the bulky layer modeled by a slab of thickness *d*. The pillars are presented as truncated cones arranged in square arrays with period *a*. e–g) Adapted with permission.^[^ ^13^ ^]^ Copyright 2019, American Physical Society. h) Photograph of a *Greta oto* butterfly. Wingspan ≈ 47 mm. Reproduced with permission,^[^ ^14^ ^]^ Copyright 2009, The Royal Society of Chemistry. i) A cross‐section of the wing membrane prepared by focused ion beam and imaged by SEM. j) Experimental angle‐resolved specular reflection spectra measured on the transparent region of the glasswing butterfly for various incident angles. k) Schematics of the anti‐reflection structures used to simulate the anti‐reflection properties of the glasswing structure. First, nanopillars with fixed radius but randomly distributed height (blue, top) are introduced. Later, the model is improved by considering the pedestals of the glasswing structures by adding pedestals of height *h*_*p*_ (green, bottom). i– k) Reproduced with permission.^[^ ^15^ ^]^ Copyright 2019, Springer Nature.

In the bulk, a lowering of the refractive index contrast might lead to a shift from a multiple scattering to a ballistic transport regime (Figure 1). An example of a reversible refractive index matching is observed in the flowers of *Diphylleia grayi* functioning as a communication mechanism with pollinators.^[^ ^10^ ^]^ On dry days, the loose cell structure of the flower petals contains several air inclusions leading to light scattering and a white appearance (**Figure** **2**a). However, when the flowers are wet by rain, water penetrates the inner petal tissues, decreasing the refractive index contrasts in the petal and leading to their transparency (Figure 2b). While this phenomenon is not very common in land plants, transparency is frequently found in aquatic life where water (*n* = 1.33) decreases the overall refractive index contrast.^[^ ^11^ ^]^

Minimizing scattering, in terms of decreasing the reflection at the interface of a material, is especially important for visual organs and for camouflage (transparent structures are challenging to be seen by predators). Several mechanisms have been observed to achieve anti‐reflective structures in the eyes and wings of insects. A common one is to build multi‐layer structures with gradually changing layer thickness and alternating refractive indexes by using multiple materials. This approach minimizes reflection in the back‐scattering direction by destructive interference of light. Similar effects can be achieved by nanostructured surfaces, which cause a gradual change in the average refractive index. A well‐known example of natural anti‐reflective coating is the surface of the moth’s eye, which is covered with nanopillars.^[^ ^16^ ^]^ Similarly, the transparent wings of the hawkmoth *Cephonodes hylas* are covered with nanopillar arrays with a very regular spacing of 200 nm in an ordered hexagonal arrangement showing high accuracy in height (250 nm)^[^ ^12^ ^]^ (Figure 2c,d). Most pillar‐like anti‐reflective coatings found in nature feature periodic arrangements with constant height and aspect ratio of 1–2.^[^ ^15^ ^]^

However, periodicity and close packing is not a requirement to achieve anti‐reflective properties. The transparent wings as those of the moth *Cacostatia ossa* (Figure 2e,f) show non‐close‐packed rod‐like arrays.^[^ ^13^ ^]^ In this study, a 3D transfer matrix model based on a detailed morphological characterization (Figure 2g) is used to explain a reduction of the reflection from 5% to less than 1%. The cone shape of the nanostructures causes a smooth impedance match between cuticle and air. While the single cone is not detectable by visible light due to its small size, the array of nanostructures acts as a homogeneous transparent film in *x*, *y* direction with an inhomogeneous change of the refractive index in *z*. The cones can be considered as a continuous layer having an effective refractive index gradually changing from the outer air to the corneal tissue.

Omnidirectional broadband antireflection has been observed in *Greta oto* butterfly wings (Figure 2h) with disordered arrangement and size distribution of nanopillars (Figure 2i).^[^ ^14^, ^15^ ^]^ The angle independency of the broadband antireflection is characterized experimentally via angular resolved specular reflection measurements (Figure 2j). This outstanding optical property is explained by a model combing an effective index theory with the transfer matrix method. At the origin of the omnidirectional antireflection properties is the random distribution of height and width of the nanopillars (Figure 2k) rather than their random arrangement. A further example of transparency using disordered surfaces in nature is found in the wing membrane of the dragonfly *Aeshna cyanea*, where a rough surface of wax structures was observed.

Besides anti‐reflection properties, natural nanostructures have multi‐functional properties as they are often also hydrophobic. In flying insects, such a feature is highly desirable to avoid carrying the extra weight of water. As an example, the wings *of Cicada Orni* have been shown to achieve both super‐hydrophobic and anti‐reflective properties employing a two‐level system of nanopillars.^[^ ^17^ ^]^ On the lower level, the pillars are forming truncated cones, again acting as an anti‐reflection film with gradually changing effective refractive index. The top is covered with a hemisphere not affecting the anti‐reflection properties that instead provide super‐hydrophobicity.

### Maximized Scattering: Whiteness in Biological Systems

As illustrated in Figure 1, the amount of light scattered by a system depends on the interplay of single‐particle and ensemble properties. The easiest parameter to control, and arguably also the most crucial in scattering optimization, is the refractive index of the particles. Despite being composed mainly of biopolymers, whose refractive indices are typically restricted in a small range of values around *n*_*bio*_ ≃ 1.5,^[^ ^23^ ^]^ natural organisms developed alternative strategies to produce whiteness such as optimizing morphology of the scatterers and introducing absorption.

**Figure 3:**
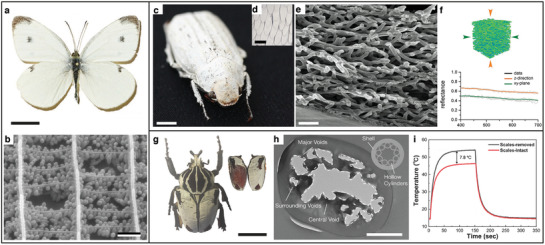
Examples of different highly scattering, white systems in nature. a) Photograph of a *Pieris rapae* butterfly. b) SEM image of the organization inside the scales covering the butterfly’s wings: Each scale contains numerous ellipsoidal particles, which are responsible for the bright, white appearance of the *P. rapae* butterfly. Scale bars: a) 1 cm, b) 1 µm. a) Adapted with permission.^[^ ^18^ ^]^ Copyright 2016, Wiley‐VCH. b) Adapted with permission.^[^ ^19^ ^]^ Copyright 2004, the Royal Society. c) Photograph of a *Cyphochilus* beetle. d) Microscopy image of the scales covering the white beetle exoskeleton. e) SEM image showing the chitin network inside each scale. Scale bars: c) 1 cm, d) 200 µm, e) 1 µm. c–e) Adapted with permission.^[^ ^20^ ^]^ Copyright 2018, The Authors, published by the Royal Society. f) 3D reconstruction of the chitin network in the *Cyphochilus*’ scales and simulated reflectance along two (the chitin network is isotropic in the *xy* plane) perpendicular directions of the incident light. A high reflectance (≃70%) is observed with the extremely small thickness of the scales (≃7 μm). The optical anisotropy is a consequence of the preferential alignment direction of the chitin fibers perpendicular to the scale’s interface. Adapted under the terms of the CC‐BY Creative Commons Attribution 4.0 license (https://creativecommons.org/licenses/by/4.0/).^[^ ^21^ ^]^ Copyright 2017, The Authors, published by Wiley‐VCH. g) Photograph of a *Goliathus* beetle. h) Transmission electron microscopy (TEM) image of a tubular *Goliathus*’ scale from a cross‐sectional view. The inset represents a simplified, schematic representation of the cross‐sectional view of a scale. i) Temperature profiles in function of the exposure under a xenon lamp, measured for beetle elytra with and without scales. The white scales limit the temperature increase and allow the *Goliathus* beetle to survive under long sunlight exposure. Scale bars: g) 5 cm, h) 500 nm. g–i) Adapted with permission.^[^ ^22^ ^]^ Copyright 2019, The Royal Society of Chemistry.

An example of a carefully engineered interplay between scattering and absorption for whiteness production is observed in the butterfly species *Pieris rapae* (**Figure** **3**a). As shown in Figure 3b, the scales covering the butterfly’s wings contain a disordered ensemble of elliptical particles made of pterin pigment and embedded in a keratin matrix. While the wing scales have an average refractive index of 1.75–1.9, the individual particles achieve a refractive index larger than 2 over the whole visible range by exploiting the intrinsic connection of the real part of the refractive index with its imaginary part that dictate absorption (cf. Kramers–Kronig relations). More in detail, the pterin pigment inside each particle is characterized by a strong absorption below 400 nm. This absorption causes the high value of refractive index for low visible wavelengths. The scattering is further enhanced by the optimized elliptical shape of the particles. Numerical simulations demonstrated that by fixing the average volume of a particle, the anisotropic shape leads to an increase of the scattering efficiency.^[^ ^18^ ^]^

Anisotropy is a key feature also for another striking example of scattering optimization in nature: the beetle genus *Cyphochilus* (Figure 3c). The white appearance of this insect is achieved by the chitin, disordered network inside the scales covering its exoskeleton (Figure 3d,e). This sub‐micrometer anisotropic network is particularly interesting for the study of disordered systems, as it exhibits the lowest transport mean free path observed in biological materials thus far. The optical properties of the beetle genus *Cyphochilus* were first studied in 2007 by Vukusic et al.^[^ ^24^ ^]^ The exoskeleton of this white beetle is covered by a single layer of scales, (Figure 3c,d) whose dimensions are about 7 µm in thickness, 250 µm in length, and 100 µm in width.^[^ ^25^ ^]^ As shown in Figure 3e, and further demonstrated by the 3D reconstruction performed by Wilts et al.,^[^ ^21^ ^]^ each scale contains a disordered network of fibers with an anisotropic alignment, that is, mainly oriented parallel to the surface of the scales. The filling fraction of this network has been reported to be 45 %, while the average radius and length of the fibers are (0.12 ± 0.08) μm and (1.1 ± 0.4) μm, respectively.^[^ ^21^ ^]^

The striking scattering strength of the *Cyphochilus* scales originated a long debate on the experimental characterization and theoretical understanding of light transport in this disordered, anisotropic system. From an experimental point of view, the mean free path in the direction perpendicular to scales’ surface (ℓ_*z*_) was first estimated by Burresi et al. to be between 0.9 and 1.6 µm.^[^ ^26^ ^]^ This value is smaller than the in‐plane component (ℓ_*xy*_) and represented the smallest value ever reported for a low‐refractive‐index system.^[^ ^27^ ^]^ The anisotropy in the mean free path, that is shown in Figure 3f in terms of anisotropy in the reflectance, is a consequence of the orientational anisotropy in the beetle’s scales and it suggests that the chitin disordered network is optimized to increase scattering along the direction perpendicular to the surface of the scales.^[^ ^26^, ^27^ ^]^ Although these early experimental results demonstrated the uniqueness of the optical properties of the *Cyphochilus* beetle, the accuracy in determining the different components of the mean free path was limited by the strong thickness dependency of the experimental techniques used, namely photon lifetime^[^ ^26^ ^]^ and total transmission^[^ ^27^ ^]^ measurements. In this biological sample, the thickness varies from scale to scale, therefore limiting the accuracy of the aforementioned experimental approaches. This limitation has been recently overcome by combining a measurement of the coherent backscattering phenomenon with anisotropic Monte Carlo simulations,^[^ ^6^ ^]^ providing a thickness‐independent value for the in‐plane component and more accurate estimate of the out‐of‐plane component of the mean free path: ℓ_*xy*_ = (1.4 ± 0.1) µm and ℓ_*z*_ = (1.0 ± 0.2) µm, respectively.

Understanding the mechanism behind the extremely high scattering efficiency observed in the *Cyphochilus* beetle is of crucial importance for reproducing similar optical performances in artificial, low‐index materials. A first attempt in understanding light propagation in the scales of the Cyphochilus was performed by Wilts et al. using image manipulation on the 3D reconstruction of the chitin network.^[^ ^21^ ^]^ However, such an approach did not allow to control the orientation of the scattering elements and to independently vary the crucial parameters for scattering, namely the shape and the size of the scattering elements as well as thickness and filling fraction of the network. Different numerical algorithms have been recently developed to separate these contributions and gain insight into the scattering optimization process.^[^ ^6^, ^7^, ^28^ ^]^ By exploring a large parameter space for single‐particle and ensemble configurations, Jacucci et al. demonstrated that optimized ensembles of anisotropic scatterers outperform the scattering efficiency of their isotropic counterpart.^[^ ^6^ ^]^ In detail, this result is valid when the anisotropic system exhibits an orientational order where the long axis of the scatterers is on average perpendicular to the incoming beam,^[^ ^6^, ^7^ ^]^ as showcased by the *Cyphochilus* beetle. Moreover, these numerical studies indicated the optimal filling fraction for beetle‐like anisotropic systems to be around 30 %. Interestingly, this result corresponds to the value of filling fraction for the *Cyphochilus* network recently reported by Burg et al.^[^ ^28^ ^]^ In this study, a non‐destructive, 3D x‐ray nanotomography was used to reconstruct the morphology of the chitin network. This technique is quantitatively more accurate than the previously reported ones as possible artifacts arising from sectioning the scales are avoided.

Although the scattering properties of the *Cyphochilus* beetle have been extensively examined, their biological role is still not clear. A recent work on the beetle genus *Goliathus* (Figure 3g) demonstrated that its white appearance might play a key role in lowering the insect’s temperature via radiative cooling.^[^ ^29^, ^30^, ^31^ ^]^ Radiative cooling is the phenomenon through which a material decreases its temperature by exchanging energy with the upper atmosphere or outer space. This latter happens when a material emits thermal radiation at wavelengths where the atmosphere is transparent and efficiently reflects sunlight to avoid counteracting heating effects. The *Goliathus* beetle exploits a combination of thin‐film interference and Mie resonances (i.e., resonances in the scattering cross‐section of a particle^[^ ^1^, ^5^ ^]^) to maximize the reflection over the visible range. As shown in Figure 3h, the *Goliathus*’ exoskeleton is covered with tubular scales whose internal structure consists of voids uniformly distributed around the outer chitin cortex. By describing this disordered system as a shell/hollow cylinder structure, Xie et al. demonstrated that the white appearance of this insect is achieved by optimizing the number and size of voids in each scale. Also, the shell/hollow cylinder morphology contributes to an increase in the mid‐infrared (MIR) emissivity of the scales, resulting in an effective self‐cooling mechanism. Figure 3i shows that as a consequence of radiative cooling, the presence of the white scales reduces the increase of temperature under light exposure of ≃7.8° C. As in the case of the *Goliathus* beetle, but employing different structures, radiative cooling has been recently observed in other organisms.^[^ ^32^, ^33^ ^]^

## Mimicking Nature with Biomaterials

In this section, we summarize some of the strategies exploited to maximize or minimize light scattering in cellulose‐based artificial materials.

### Light Management in Cellulose Materials

While nature can be a great inspiration for designing optical appearances (cf. Section 2), it can also be a convenient resource of raw materials. Many different biopolymers have been exploited to produce photonic structures. In this context, cellulose is particularly advantageous: not only for its abundance and intrinsic renewability,^[^ ^34^ ^]^ but also for its high refractive index and birefringence values compared to other biopolymers (*n* ≈ 1.5, Δ*n* ≈ 0.074 − 0.08).^[^ ^35^ ^]^

**Figure 4:**
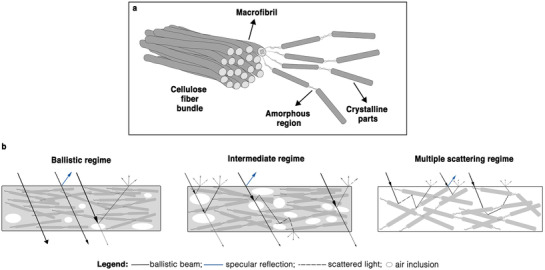
Cellulose and its use for photonic applications. a) Illustration of the hierarchical architecture of a cellulose fiber. b) Illustration of different scattering regimes in cellulose‐based materials. The transition from different regimes is represented by an increasing number of air inclusions, which increases scattering. The ballistic beam, specular reflection, and scattered light are represented by the solid black, solid blue, and dotted black lines, respectively. The line thickness qualitatively represents the difference in intensity between the ballistic and the scattered light in every regime.

Cellulose constitutes the major component of the plant cell wall and, in nature, is found in a hierarchical fibrillar form, which consists of macrofibrils that are composed of microfibrils. These microfibrils are formed from β‐1,4‐glucan chains, which are organized in amorphous and crystalline regions (**Figure** **4**a). Macroscopic cellulose fibers are an essential resource for papermaking and the production of cellulose‐based polymers.^[^ ^36^ ^]^ The recent advancement in nanotechnologies has attracted massive attention to the exploitation of cellulose for its unique chemical and physical properties at the nano‐scale. Nanocellulose is a term referring to cellulosic fibrillar particles extracted from natural cellulose with at least one dimension in the nanometer range (1–100 nm).^[^ ^37^ ^]^ Nanocelluloses can be classified into two categories based on their dimensions: cellulose nanofibrils (CNFs)^[^ ^38^ ^]^ and cellulose nanocrystals (CNCs).^[^ ^39^ ^]^ CNFs usually have a width of about 3–20 nm, a length of few micrometres, and can be extracted from the defibrillation of cellulose fibers by enzymatic,^[^ ^40^ ^]^ or chemical pre‐treatment (including 2,2,6,6‐tetramethylpiperidine‐1‐oxyl radical [TEMPO],^[^ ^41^ ^]^ or periodate‐chlorite oxidation^[^ ^42^ ^]^) followed by mechanical treatment. In contrast, CNCs are chemically extracted from cellulose fibers by hydrolysis with a strong acid (usually sulfuric acid),^[^ ^43^ ^]^ and they are rod‐shaped crystalline particles with a lateral size of about 3–5 nm and much shorter length (100–200 nm) compared to CNFs.

#### Transparent Films from Cellulose Fibers

As discussed for the case of living organisms, bulk transparency can be achieved by homogeneous, non‐absorbing materials. While it is relatively simple to achieve transparent polymer films if the constituting polymer can be dissolved entirely and precipitated from a solvent, it is challenging to obtain transparency when starting from natural fibers extracted from plants with limited chemical modification. To achieve transparency, the fibers need to be packed efficiently into films, and porosity needs to be very low. As schematically illustrated in Figure 4b, porosity induced by insufficient packing between nanofibrils can induce scattering. The size and distribution of the air pores inside the material can lead to different scattering regimes: from ballistic (where air inclusions are isolated and small) to intermediate to multiple scattering (where the porosity is so high so that the scattering elements become the fibers themselves). As scattering is a bulk phenomenon, it is important to highlight that the thickness also plays a crucial role—as the fundamental parameter to consider is the optical thickness (cf. Section 1).

**Figure 5:**
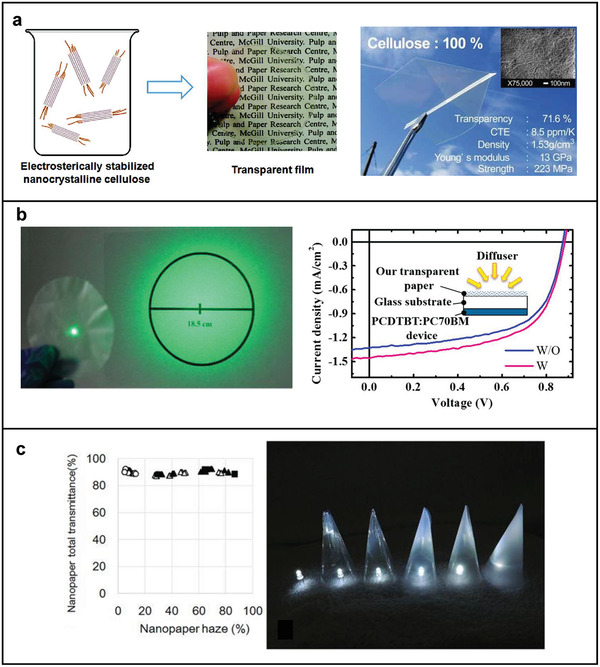
Examples of different artificial transparent films. a) Schematic of the highly charged CNCs, which have a large quantity of di‐ and tricarboxylated cellulose chains protruding from their surface and providing electrosterical stability, and a picture of the transparent film prepared from highly charged CNCs by vacuum filtration (left). Adapted with permission.^[^ ^44^ ^]^ Copyright 2012, American Chemical Society. Transparent film fabricated from CNFs exhibits high Young’s modulus, high strength, ultralow CTE, and high foldability. Inset is the SEM image of the surface structure of the transparent film; the scale bar is 100 nm (right). Adapted with permission.^[^ ^45^ ^]^ Copyright 2009, Wiley‐VCH. b) A picture showing the light‐scattering effect of the transparent film when a laser with a diameter of 0.4 cm passes through it; a larger illuminated circular area with a diameter of over 18.5 cm was formed on the target (the distance between the transparent film and target is about 30 cm) (left). Comparison of the *I–V* curves of the organic photovoltaic (OPV) device illuminated by diffused light (right), W and W/O represent OPV device with and without transparent film, respectively. Adapted with permission.^[^ ^46^ ^]^ Copyright 2013, American Chemical Society. c) Graph (left) showing the total light transmittance of nanopaper can be maintained and that the haze value can be adjusted in a wide range, and a picture (right) showing the application of the hazy transparent nanopaper as optical diffusers: an LED light without a diffuser, and with increasing haze of nanopaper, which homogeneously spreads the light through the nanopaper and maintains light intensity. Adapted under the terms of the CC‐BY Creative Commons Attribution 4.0 International License (http://creativecommons.org/licenses/by/4.0/).^[^ ^47^ ^]^ Copyright 2017, The Authors, published by Springer Nature

While CNCs would allow achieving better packing efficiency, the mechanical properties of CNC films are generally poor. CNC films are, in fact, quite brittle due to the crystalline nature of CNCs.^[^ ^48^ ^]^ The mechanical properties of CNC‐based transparent films were improved by using highly charged CNCs (HCNCs) obtained from sequential periodate‐chlorite oxidation of wood fibers.^[^ ^49^ ^]^ HCNCs have a width of about 10 nm and a length of 90–220 nm. The large number (3.5 mmol g^‐1^) of carboxylic acid groups on their surface enable cross‐linking between HCNCs during solvent removal, leading to improved mechanical performance. **Figure** **5**a (left inset) shows these 30‐µm‐thick HCNC films with a transmittance of about 87%.^[^ ^44^ ^]^

In contrast, CNFs can be used to produce transparent films while maintaining their mechanical stability.^[^ ^50^ ^]^ The first example of a 55‐µm‐thick CNF transparent film has been obtained using CNFs with an average diameter of 15–20 nm.^[^ ^45^ ^]^ As shown in Figure 5a (right inset), the resulting films show a transmittance of 71.6%, and they have high Young’s modulus (13 GPa), high tensile strength (223 MPa), and low coefficients of thermal expansion (CTEs ≃ 8.5 ppm K^–1^). These properties make them an excellent substrate material for electronic devices compared to current alternatives (such as glass or flexible plastics: that are either brittle or have high CTEs, respectively).

CNFs with a smaller diameter (of about 3–4 nm) were used to produce films with improved transmittance reaching a value of about 90% (20 µm thickness).^[^ ^51^ ^]^ In this case, CNFs were prepared from TEMPO‐oxidized softwood cellulose. The high transmittance is due to the smaller diameter of the CNFs and the improved fiber‐to‐fiber interactions (hydrogen bonding and capillary force) during drying process.^[^ ^51^, ^52^ ^]^ Such interactions allow CNFs to pack more tightly. As a consequence, both the reduced inhomogeneities and smooth film surface contribute to minimizing light scattering.^[^ ^53^ ^]^ However, the better optical performances of CNFs with smaller size come at the cost of the mechanical strength of the films.

Nanocelluloses and wood‐based fibers have also been exploited to fabricate films in the intermediate scattering regime with simultaneously optimized optical haze and transmission. Optical haze is the ratio of the transmitted light that is diffusely scattered (the forward‐scattered light) to the transmitted light. High optical haze is desirable for many optoelectronic applications: For instance, substrates with high optical haze can enhance the efficiency of light‐harvesting in solar cells,^[^ ^57^ ^]^ and can help remove glare for outdoor displays.^[^ ^53^, ^58^ ^]^ However, achieving simultaneously high transparency and haze is challenging as these two properties are usually connected. Fang et al. reported films with ultrahigh transmittance (about 96%) and high optical haze (about 60%) obtained with TEMPO‐oxidized wood‐derived fibers without any mechanical treatment.^[^ ^46^ ^]^ This cost‐effective strategy resulted in a highly stacked microfiber network, where the high haze is the result of the refractive index mismatch between cellulose and air inclusions. The optical effect of haze and its beneficial properties for optoelectronic applications are shown in Figure 5b.^[^ ^46^ ^]^

The optical haze of CNF‐based films can be tailored in a wide range from 27% to 86% while maintaining high total transmittance (around 90%) by changing the content of the micro‐sized fiber fragments, which are responsible for introducing air inclusions (Figure 5c, left inset).^[^ ^47^ ^]^ The hazy, transparent CNF films obtained with this technique scatter the incident light without strongly affecting the transmission, and can be exploited as optical diffusers for LED (Figure 5c, right inset).

Films with high haze have also been produced by hot‐pressing of cellulose microfibers, which have been partially dissolved by the ionic liquid^[^ ^53^ ^]^; or by incorporation of polymers via refractive index matching; as an example, hazy all‐cellulose composite films were obtained by filling hazy paper of uniformly distributed cellulose fiber network with index‐matched carboxymethyl cellulose polymer.^[^ ^59^ ^]^

#### White Materials from Cellulose Biopolymers and Fibers

As reported in Section 2.2, anisotropic porous structures are ideal for maximizing multiple scattering for the production of white materials. The ability to control and improve scattering efficiency using only natural materials is extremely important in applications such as paints, coatings, and white enhancers. It is crucial to avoid the use of current whitening agents, such as carcinogenic titanium dioxide nanoparticles, especially for cosmetic and food coloration. In this context, highly porous cellulose materials have been shown to be ideal candidates. In the following, two recent examples of engineered scattering materials based on cellulose nanocrystals (CNCs) and cellulose nanofibrils (CNFs) are introduced.

**Figure 6:**
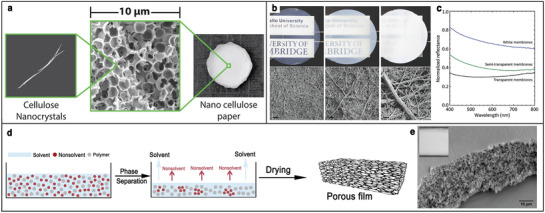
Examples of different artificial highly scattering, white materials. a) Photograph of the nanocellulose paper. SEM image showing the internal morphology of paper and the cellulose nanocrystals. Adapted under the terms of ACS AuthorChoice/Editors’ Choice via Creative Commons CC‐BY Usage Agreement (https://pubs.acs.org/page/policy/authorchoice_ccby_termsofuse.html).^[^ ^54^ ^]^ Copyright 2017, American Chemical Society. b) Photographs of the CNF films using, from left to right: the finest, the medium, and the coarsest fibrils. Top surface SEM images of the transparent, semitransparent, and white membranes. Scale bar: 1 µm. c) Total reflectance spectra of the three membranes at a thickness of 9 µm. b,c) Adapted under the terms of CC‐BY Creative Commons Attribution 4.0 License (https://creativecommons.org/licenses/by/4.0/).^[^ ^55^ ^]^ Copyright 2018, The Authors, published by Wiley‐VCH. d) Representative phase separation process for the fabrication of porous films. Adapted under the terms of CC‐BY Creative Commons Attribution 4.0 International license (https://creativecommons.org/licenses/by/4.0/).^[^ ^56^ ^]^ Copyright 2018, The Authors, published by Wiley‐VCH. e) SEM image of the porous cellulose acetate film, with an inset showing the white appearance. Adapted under the terms of CC‐BY Creative Commons Attribution 4.0 International license (https://creativecommons.org/licenses/by/4.0/).^[^ ^28^ ^]^ Copyright 2019, The Authors, published by Springer Nature.

Caixeiro et al. were the first to use CNCs for optimizing multiple scattering. In this work, an inverse photonic glass architecture was used to achieve the required porosity to enter the multiple scattering regime in the film.^[^ ^54^ ^]^ **Figure** **6**a shows the SEM image of the fabricated film, in which a 3D disordered close‐packed pore configuration is visible. The nanostructured films achieved with this method exhibit a mean free path value four times smaller than that of standard cellulose filter paper (**Table** **1**), implying that the same scattering efficiency can be achieved with a much smaller amount of material.

**Table 1:** Transport mean free path at 500 nm wavelength for different natural and artificial white materials

Material	ℓ_*t*_ [µm]
CNC paper^[^ ^54^ ^]^	≃4
*Cyphochilus* beetle^[^ ^26^ ^]^	1.47 ± 0.07
Paper^[^ ^26^ ^]^	13 ± 0.65
PS films^[^ ^60^ ^]^	0.98 ± 0.03
PMMA films^[^ ^56^ ^]^	≃1

The other example is based on the fact that higher scattering strength can be achieved using a network morphology. Toivonen et al.^[^ ^55^ ^]^ produced CNF porous membranes of different scattering properties by solvent‐exchange method, which is achieved with CNFs in different diameters separated from sequential centrifugation. As depicted in Figure 6b, by changing the morphology of the membrane (in terms of porosity and fibrillar morphology), it is possible to achieve different appearances: from transparent to white. The white membranes produced with this technique exhibit a reflectivity of around 60–80% over the visible light range while being 9 µm thick.

Another strategy to produce highly scattering material based on fibrillar‐like networks is to exploit polymer phase separation combined with kinetic arrest. A general phase separation process is shown in Figure 6d. While this strategy has been largely used to produce different type aerogel materials,^[^ ^61^ ^]^ the morphology of such system have never been optimized to maximize scattering. Such method has been exploited to produce highly scattering materials with cellulose derivatives,^[^ ^28^ ^]^ as cellulose acetate shows in Figure 6e, and several commercially available polymers.^[^ ^56^, ^60^ ^]^ The scattering efficiency of some of these artificial systems is compared in Table 1 in terms of the mean free path (ℓ_*t*_, cf. Section 1).

### Transparent and White Wood

**Figure 7:**
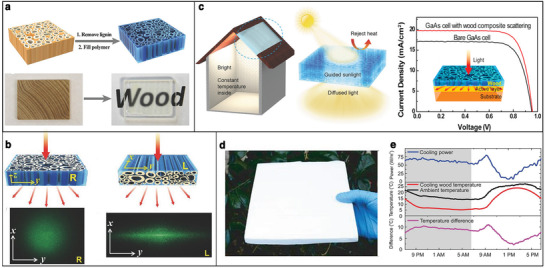
Examples of different artificial transparent and white wood. a) Schematic of the mesoporous structures in the wood where the cell walls are aligned in the same direction as the tree growth, after lignin is removed and the index‐matching polymer is filled in (top). Pictures (below) showing that a thick (up to centimeter) piece of wood becomes highly transparent after the two‐step treatment. b) Schematic of the transmittance measurement setup for transparent wood with two different anisotropic structures, and pictures of the scattered light spot for transparent wood with two different anisotropic structures, respectively. a,b) Adapted with permission.^[^ ^62^ ^]^ Copyright 2016, Wiley‐VCH. c) Schematic of an application of transparent wood used in a house as rooftop, which can efficiently harvest and guide sunlight to achieve a comfortable and uniform lighting condition and maintain a constant ambient temperature inside (left). Reproduced with permission.^[^ ^63^ ^]^ Copyright 2016, Wiley‐VCH. Graph of current density versus voltage characteristics for both bare GaAs cell and the GaAs cell with a light‐management wood coating (inset: schematic of light distribution incident on a solar cell) (right). Reproduced with permission.^[^ ^64^ ^]^ Copyright 2016, Elsevier. d) Photograph of the cooling wood. e) 24 h continuous measurement of the cooling power, steady‐state temperature, and temperature difference compared to the ambient surroundings of the cooling wood. d,e) Adapted with permission^[^ ^65^ ^]^ Copyright 2019, The Authors, published by The American Association for the Advancement of Science.

Interestingly, the same concept used to design transparent or highly scattering materials can be extended to wood matrices. The first work on transparent wood was achieved by infiltrating the chemically bleached wood with an index‐matching polymer,^[^ ^66^ ^]^ as the same mechanism of index matching for flower petals reported in Figure 2b. As shown in **Figure** **7**a, natural wood appears brownish and non‐transparent in the visible range because of the strong absorption from lignin and the scattering of light from its inhomogeneous architecture. Such absorption can be greatly reduced by chemical removal of lignin with sodium hypochlorite,^[^ ^66^ ^]^ sodium chlorite,^[^ ^67^ ^]^ or sodium hydroxide/sodium sulfite.^[^ ^62^, ^63^ ^]^ This process is called delignification. Alternatively, alkaline hydrogen peroxide treatment only deactivates the chromophores in the lignin, so this method allows to reduce absorption while better preserving the mechanical properties of the wood.^[^ ^68^ ^]^

Achieving perfect index matching of wood is challenging, as the polymers infiltrated into the wood matrix need to: 1) be transparent, 2) show little shrinkage during polymerization, and more importantly 3) have the same refractive index of the matrix (*n* ≃ 1.53).^[^ ^62^, ^63^, ^64^, ^68^ ^]^ When the mismatch between the refractive index of wood and the infiltrated polymer is small, the scattering is greatly reduced, thus forming a transparent wood. Transmittance can be increased by improving the compatibility between wood template and polymers. For example, acetylation of the wood template eliminates the interface debonding gap between the filled polymers and cellulose inner walls.^[^ ^69^ ^]^

Zhu et al. reported that mechanical compression of delignified wood can also lead to reduced scattering. This method has the advantage of achieving transparent wood without using any index‐matching polymer.^[^ ^70^ ^]^ Mechanical compression allows to densify the delignified wood and reduces the porosity, therefore decreases the scattering strength of the wood. However, while for compressed delignified wood with small thickness, the material appears transparent, for a few centimeters thickness, the light transport is not ballistic, and the material becomes opaque. Finally, as wood is intrinsically anisotropic structure, the haze properties can be manipulated by cutting the wood in different directions, as shown in Figure 7b. In fact, light scattering is isotropic along the plane perpendicular to the wood trunk (R‐wood), while it is highly anisotropic along the plane parallel to the direction of growth (L‐wood).^[^ ^62^ ^]^

Transparent wood is a promising, environmentally friendly, and energy‐efficient building material, due to its outstanding optical properties, low thermal conductivity, and excellent mechanical properties. As shown in Figure 7c (left inset), a house with a transparent wood rooftop can achieve more uniform lighting, since it can guide the sunlight transmittance along with the wood cell and largely scatter it in the forward direction.^[^ ^63^, ^64^ ^]^ Furthermore, transparent wood has better impact strength and lower density than glass; by functionalization with photochromic materials, it can be used as photo‐switchable smart window.^[^ ^71^ ^]^ Transparent wood also can improve the energy conversion efficiency as coatings for solar cells by increasing the light absorption (Figure 7c, right inset).^[^ ^64^ ^]^ However, it is still challenging to fully transfer these applications to industrial products as the fabrication processes for large scale transparent wood are still lacking.

In contrast, white wood can be easily obtained by simple delignification. In fact, even if the scattering properties of the wood fibers are not optimized, the large volume increases the number of scattering events to make the material white. Radiative cooling has been recently achieved using such structure: Li et al. developed a structural material called cooling wood as shown in Figure 7d, which is produced by complete delignification and partial densification of wood.^[^ ^65^ ^]^ Figure 7e shows its cooling power and the temperature difference with the ambient environment.

## Summary and Outlook

In summary, materials with a tailored scattering response, such as maximum scattering strength (whiteness) or transparency, are essential for next‐generation lightening devices and coatings, but also crucial in studying fundamental light transport effects in complex media. Despite significant steps made in the fabrication of biopolymer‐based products, we believe that there is still space for improvement, especially at the two extreme regimes of transport.

Fully transparent nanocellulose films combining simultaneously excellent optical and mechanical properties are still missing. At small fibril diameters, the optical response is improved, but the mechanical properties worsen. While this challenges the preparation, we believe the appropriate mixture of CNCs with CNFs could lead to films with improved mechanical performance while maintaining good transparency. It has been demonstrated that by adding a minor fraction of tunicate CNCs (aspect ratio is about 100, the length is about 1000 nm), all CNC‐based films can show remarkable improvement in mechanical performance.^[^ ^72^ ^]^ Finally, chemical modification methods can also be explored to improve the response of CNC‐based transparent films: such as by physically (by adsorption) or chemically (by covalent bonds) attaching/grafting long‐chain surfactants^[^ ^48^ ^]^ or polymers^[^ ^73^ ^]^ on the CNCs.

In the case of whiteness, nanocellulose allowed to obtain highly scattering materials with scattering strengths comparable to their biological counterparts. However, the intrinsic anisotropy of the cellulose nanofibers and careful optimization of their size have not been explored. Similarly, large volume methodologies for the fabrication of highly scattering material that can be exploited in commercial applications are still missing. Finally, nature can still serve as inspiration for such types of materials. As an example, exploiting the relation between the imaginary and the real part of the refractive index, as in the case of *P. Rapae* butterfly, can lead to a further increase in the scattering efficiency of bio‐inspired systems.

## Conflict of Interest

The authors declare no conflict of interest.
